# Targeted DNA sequencing of non-small cell lung cancer identifies mutations associated with brain metastases

**DOI:** 10.18632/oncotarget.25409

**Published:** 2018-05-25

**Authors:** George D. Wilson, Matthew D. Johnson, Samreen Ahmed, Paola Yumpo Cardenas, Inga S. Grills, Bryan J. Thibodeau

**Affiliations:** ^1^ Department of Radiation Oncology, William Beaumont Hospital, Royal Oak, MI, USA; ^2^ Beaumont BioBank, William Beaumont Hospital, Royal Oak, MI, USA; ^3^ Cancer Genetics Program, William Beaumont Hospital, Royal Oak, MI, USA; ^4^ Department of Radiation Oncology, McLaren Health Care, Macomb, MI, USA

**Keywords:** non-small cell lung cancer, brain metastases, next generation DNA sequencing, targeted agents, radiotherapy

## Abstract

**Introduction:**

This study explores the hypothesis that dominant molecular oncogenes in non-small cell lung cancer (NSCLC) are associated with metastatic spread to the brain.

**Methods:**

NSCLC patient groups with no evidence of metastasis, with metastatic disease to a non-CNS site, who developed brain metastasis after diagnosis, and patients with simultaneous diagnosis of NSCLC and metastatic brain lesions were studied using targeted sequencing.

**Results:**

In patients with brain metastasis versus those without, only 2 variants (one each in BCL6 and NOTHC2) were identified that occurred in ≥ 4 NSCLC of patients with brain metastases but ≤ 1 of the NSCLC samples without brain metastases. At the gene level, 20 genes were found to have unique variants in more than 33% of the patients with brain metastases. When analyzed at the patient level, these 20 genes formed the basis of a predictive test to discriminate those with brain metastasis. Further analysis showed that PI3K/AKT signaling is altered in both the primary and metastases of NSCLC patients with brain lesions.

**Conclusion:**

While no single variant was associated with brain metastasis, this study describes a potential gene panel for the identification of patients at risk and implicates PI3K/AKT signaling as a therapeutic target.

## INTRODUCTION

Lung cancer is the most common cause of cancer-related mortality in the United States and worldwide where there are over 1.5 million deaths each year [1]. Non-small cell lung cancer (NSCLC) is the most common lung cancer (>85% of all lung cancers), and adenocarcinoma is the most common histology within NSCLC. Adenocarcinomas have a propensity to metastasize to the central nervous system (CNS) [2], and it is estimated that up to 40% of patients will develop a brain metastasis (BM) spread [3, 4]. Unfortunately these patients have a poor prognosis which ranges from 2 months if treated symptomatically with glucocorticoids to 14 months if treated with radiation and/or neurosurgical resection [5]. Systemic therapy has not made a major impact on BMs with platinum-based therapy resulting in response rates of 28-45% in the metastatic setting [6] whilst temozolomide has demonstrated only modest effects [7].

The ability to predict which patients with primary adenocarcinomas of the lung at the time of diagnosis that are likely to metastasize to the CNS is of great importance as the incidence of CNS metastases is rising due to an aging population, better systemic treatment and increased CNS screening associated with cognitive warning signs. Several attempts have been made to develop nomograms based on clinicopathological and biochemical factors [8–10]. The nomograms have generally included parameters such as histological type, differentiation status, pN stage, pT stage, smoking status, number of metastatic lymph nodes in the mediastinum and tumor markers such as neuron-specific enolase and CA-125. These nomograms have been developed to identify the subset of patients who could be offered prophylactic cranial irradiation (PCI). Although PCI has become an integral part of the standard of care in small cell lung cancer (SCLC), its role in NSCLC remains controversial [11–13] even though previous studies have shown that PCI is able to reduce the occurrence of brain metastases by half [14, 15].

As conventional therapies fail to improve the outcome of patients with BM spread, there is hope that understanding the genetic alterations in NSCLC that lead to metastatic spread will uncover new methods to identify patients at risk and discover new avenues for treatment for distant disease. This hope is exemplified in the metastatic setting by the addition of targeted therapies against epidermal growth factor receptor (*EGFR*) or echinoderm microtubule-associated protein like 4 (*EML4*)-anaplastic lymphoma kinase (*ALK*) translocations where patients who received radiation and targeted therapy had a median survival of 21 months compared with 11 months for patients who did not receive targeted therapy [16]. However, there is still a stark mortality underpinning the need to better understand the mechanisms that govern lung cancer metastasis to the brain and uncover therapeutic opportunities.

The genetic landscape of lung adenocarcinomas has recently been reported on behalf of the Cancer Genome Atlas (TCGA) [17]. 230 matched tumor and normal tissue were studied with whole-exome sequencing and uncovered a mean somatic mutation rate of 8.87 mutations per megabase. Detailed analysis of this dataset and other previously reported datasets highlighted 18 statistically significant mutated genes including *TP53, KRAS, EGFR, BRAF, PIK3CA, MET, RIT1, STK11, KEAP1, NF1, RB1, CDKN2A, SETD2, ARID1A, SMARCA4, RBM10, U2AF1* and *MGA*. This landmark report highlights the molecular heterogeneity of lung adenocarcinoma and prompts the need for further studies in sub-groups of NSCLC. One area of research that has received modest attention is the study of the genetic aberrations associated with CNS metastasis from the lung [2, 18, 19]. Several oncogenic drivers in metastasis to the CNS have been described including genetic alterations in *EGFR, ALK, ROS1, BRAF, RET, and cMET* [20]. Additional alterations include *FGFR1* amplifications, mutations in *TP53, KRAS, CDKN2A, RAS, and PIK3CA* [21, 22] as well as alterations in the cyclin-dependent kinase and the PI3K/AKT/mTOR pathways [23]. Investigations of matched primary and metastatic tumor specimens have focused predominantly on the mutational status of *EGFR* or *KRAS* [24–27]. The available data do not establish any clear correlation between *EGFR* and *KRAS* mutation status of primary lung tumors and their propensity to metastasize to the CNS. Additional studies are needed to further investigate the link between gene mutations in primary tumors and their potential for CNS dissemination [2].

The role of targeted therapy in NSCLC brain metastases has been investigated in several clinical studies focusing on EGFR-tyrosine kinase inhibitors (gefitinib, erlotinib and afatinib) and ALK rearrangement inhibitors (crizotinib and alectinib) [28] which have shown tremendous therapeutic promise. In a recent study of 76 neurosurgical lung adenocarcinoma BM specimens, next generation sequencing of 48 cancer-associated genes identified that the most commonly mutated genes were *TP53, KRAS* and *CDKN2A*, whilst other potentially druggable alterations included *EGFR, PIK3CA, BRAF* and *SMO* [29].

In this study we have investigated two related but separate questions. Firstly, can we identify a mutation signature that predicts patients with NSCLC at presentation who are at high risk of metastasizing to the brain? To address this question we have studied primary NSCLC tumors from patients with either interval brain metastases and compared them to patients with elsewhere metastases or no evidence of metastases and performed next generation DNA sequencing of a targeted panel of 160 cancer-associated genes. The second question we addressed was whether the panel of 160 cancer-associated genes could identify pathways in brain metastases compared to primary NSCLC that were exploitable for targeted therapy in combination with radiation.

## RESULTS

### Patient characteristics

Patient data is presented in Table 1. There were 19 female and 20 males included in the study. The median age of all patients was 66 years, and this did not significantly differ between males (median age 70 years) and females (median age 65 years). The distribution of males and females was equitable between groups apart from the patients with elsewhere metastases where the majority of patients were male (7 of 8). The median time to metastases in the patients with elsewhere metastases was 35.4 months whilst those patients who developed brain metastases had a median interval of 18.0 months. Patients were treated with standard-of-care which was either lobectomy or wedge resection for the majority of NSCLC; brain metastases were mostly treated by resection and stereotactic radiosurgery. Of patients with synchronous brain metastases, only the metastatic lesions were available for analysis. For patients that developed brain metastasis, the primary and matched brain metastasis was available in 7 cases.

**Table 1:** Patient's characteristics

No metastases (minimum: 5 years)							
ID	Sample Source	Sex	Age	TNM stage	Treatment	RT	
AX4748	Lung 1°	F	65	pT1aN0M0	Lobectomy	None	
AX4749	Lung 1°	F	69	pT2aN0M0	Lobectomy	None	
AX4750	Lung 1°	M	70	pT1aN0M0	Wedge resection	None	
AX4751	Lung 1°	M	79	pT2aN0M0	Wedge resection	None	
AX4752	Lung 1°	M	72	pT1aN0M0	Wedge resection	None	
AX4753	Lung 1°	F	78	pT2aN0M0	Wedge resection	None	
AX4889	Lung 1°	M	79	pT1bN0M0	Wedge resection	None	
AX4890	Lung 1°	F	81	pT2aN0M0	Wedge resection	None	
AX4891	Lung 1°	F	68	pT1bN0M0	Wedge resection	None	
AX4892	Lung 1°	F	62	pT1aN0M0	Lobectomy	None	

Subsequent non-CNS metastases							
ID	Sample Source	Sex	Age	TNM stage	Treatment	Met. site	Time to mets.
AX5034	Lung 1°	M	64	pT1aN0M0	Wedge resection	Bone	61.1
AX5033	Lung 1°	M	62	pT2aN1M0	Lobectomy + CT	Bone	6.6
AX5024	Lung 1°	M	65	cT1aN2M0	Lobectomy + CRT	Adrenal	34
AX4899	Lung 1°	M	76	pT1aN0M0	Lobectomy	Liver	67.4
AX4896	Lung 1°	F	77	pT1aN0M0	Wedge resection	Bone	13.1
AX4895	Lung 1°	M	65	pT1aN0M0	Wedge resection	Liver/Bone	19.2
AX4894	Lung 1°	M	78	pT2aN0M0	Lobectomy	Bone	36.7
AX4893	Lung 1°	M	52	pT1aN0M0	Lobectomy	Bone	39.8

Subsequent non-CNS metastases							
ID	Sample Source	Sex	Age	TNM stage	Treatment	Met. site	Time to mets.
AX4839	Lung 1°+ Brain Met	F	63	pT2bN0M0	Lobectomy + CT	Resection + SRS	19.2
AX4840	Lung 1°+ Brain Met	M	71	pT2bN0M0	Lobectomy	Resection + SRS	39.4
AX4842	Lung 1°+ Brain Met	M	70	cT1bN2M0	Lobectomy + Neoadjuvant CT	Resection + SRS	26.3
AX4843	Lung 1°+ Brain Met	M	56	pT1aN1M0	Lobectomy + CT	Resection + SRS	16.7
AX4888	Lung 1°+ Brain Met	F	63	pT2aN0M0	Lobectomy + CT	Resection + SRS	13
AX5764	Lung 1°+ Brain Met	F	58	pT1bN0M0	Lobectomy	Resection + SRS	11.6
AX5771	Lung 1°+ Brain Met	M	71	cT2N2M0	Concurrent CRT	Resection + SRS	24.9

Subsequent CNS metastases							
ID	Sample Source	Sex	Age	TNM stage	Initial Treatment	Met. treatment	Time to mets.
AX4844	Lung 1°	M	72	pT1bN0M0	Lobectomy	Resection + SRS	79
AX5770	Lung 1°	M	74	pT1bN0M0	Lobectomy	SRS	6.7
AX5768	Lung 1°	F	58	pT3N2M0	Lobectomy + CT	SRS	4.8
AX5767	Lung 1°	F	64	pT1bN0M0	Lobectomy	SRS	7.9
AX4747	Brain Met	F	67	cT1N3M0	Concurrent CRT	Resection + SRS	61.4

Synchronous CNS metastases							
ID	Sample Source	Sex	Age	TNM stage	Treatment	Radiotherapy	Other mets
AX4729	Brain Met	M	46	T1aN0M1	Met resection + CT	Post-op SRS	None
AX4730	Brain Met	F	63	T3N2M1	Met resection + CT	Post-op SRS	None
AX4731	Brain Met	M	51	T3N0M1	Met resection + CT	Post-op SRS	None
AX4732	Brain Met	F	70	T2aN2M1	Met resection + CT	Post-op SRS	None
AX4733	Brain Met	F	73	T1bN1M1	Met resection + CT	Post-op SRS	Left adrenal
AX4734	Brain Met	M	59	T2bN2M1	Met resection + CRT to lung	Post-op SRS + EBRT to lung	None
AX4735	Brain Met	F	67	T1aN3M1	Met Resection	Post-op SRS	None
AX4736	Brain Met	M	59	T3N2M1	Met resection + CT	Post-op SRS	None
AX4737	Brain Met	F	53	T2aN1M1	Met resection + CRT to lungq	Post-op SRS + EBRT to lung	None
AX4738	Brain Met	F	51	T1aN0M1	Met resection + CT	Post-op SRS	None

CT = chemotherapy, CRT= chemoradiation, SRS = stereotactic radiosurgery, EBRT = external beam radiotherapy. Sample Source: “Lung 1°” = only primary NSCLC sequenced; “Brain Met” = only brain metastasis sequenced; “Lung 1° + Brain Met” = both samples sequenced.

### Variants in EGFR

While not the primary focus of the analysis, it is important to note the mutational status of EGFR within the patient cohort. After filtering common variants, more than 77% (37 of 48) of samples contained at least 1 variant in EGFR. The high prevalence of EGFR variance in these samples was further demonstrated by filtering to include only variants that are identified as either pathogenic by American College of Molecular Genetics and Genomics (ACMG) or damaging/probably damaging by Sorting Intolerant from Tolerant (SIFT) or PolyPhen-2 function prediction. A total of 15 variants remained after this advanced filtering; this included 14 missense single nucleotide variants and 1 in-frame deletion. For NSCLC without brain metastasis, 55% (10 of 18) contained a variant; this was comprised of variants in 7 of 10 cases without any metastasis and 3 of 8 cases of NSCLC with non-CNS metastasis. For the NSCLC patients that subsequently developed brain metastasis, 75% (9 of 12) of the primary tumor samples contained an EGFR variant while the brain metastases had variants in 87% (7 of 8). Interestingly, the 10 cases that were diagnosed with synchronous brain metastasis showed variants in only 40% (4 of 10) of the brain metastasis samples. Given the high prevalence of EGFR variation, no single variant was able to differentiate between sample types.

### Primary NSCLC with subsequent brain metastasis versus NSCLC without brain metastasis

**Figure 1:**
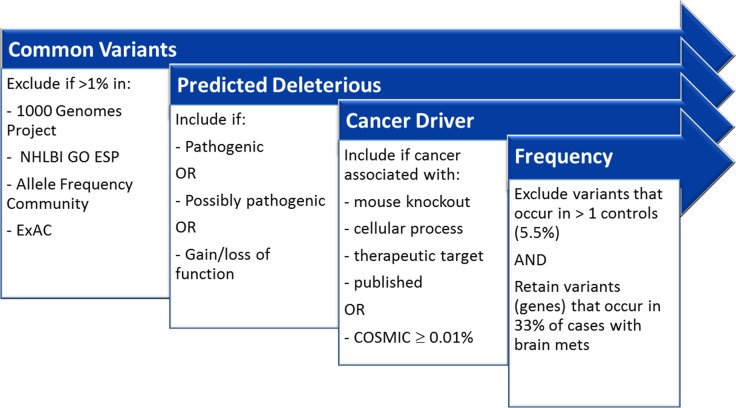
Filters implemented in Ingenuity Variant Analysis to aid in biological interpretation

The primary tumors of NSCLC patients with brain metastasis were compared to the NSCLC of patients who did not develop brain metastasis. The samples from patients with brain metastasis include only those who developed brain metastasis subsequent to NSCLC diagnosis (n=12). NSCLC patients without brain metastasis had either non-CNS associated metastasis (n=8) or had no distant metastasis (n=10). After filtering as described in Figure 1, 2 variants were identified that occurred in ≥ 4 NSCLC of patients with brain metastases but ≤ 1 of the NSCLC samples without brain metastases. A synonymous variant in NOTCH2 (c.342C>T/p.P114P) was found in 5 cases with brain metastasis while only 1 of the controls without metastasis. A missense variant in BCL6 (c.1444T>A/p.S482T) was seen in 4 of the cases with brain metastasis while no controls had the variant. Both are currently classified as variants of unknown significance by ACMG guidelines.

**Figure 2:**
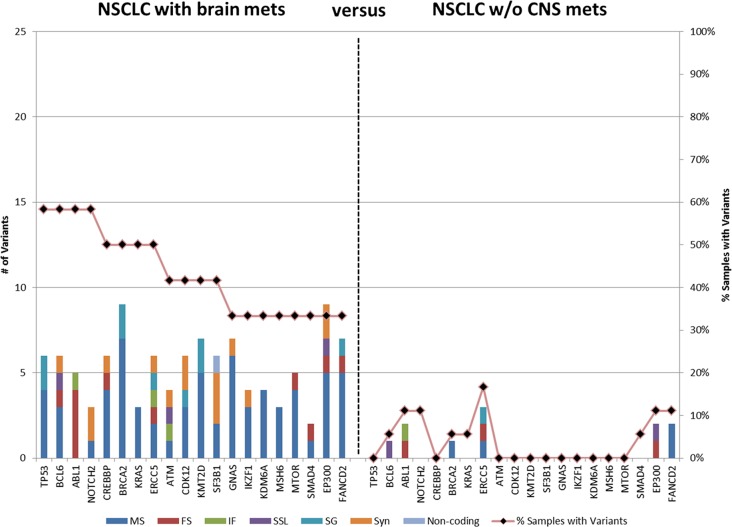
Genes containing variants in primary tumors of NSCLC patients with brain metastasis Variants in > 6% NSCLC without CNS metastasis were filtered then genes were identified with variants in ≥ 33% of primary NSCLC with brain metastasis. MS: missense, FS, frameshift; IF, in-frame; SSL, splice site loss; SG, stop gain, Syn, synonymous. The bars represent number of variants; the line represents the percentage of specimens with variants in the gene.

Filters were then employed to determine genes that have variants that differentiate the NSCLC of patients with brain metastasis. Variants that occur in > 1 of the NSCLC samples without brain metastasis were filtered (i.e. variants that occur in ≤ 6% of controls were permitted); genes were then identified that had variants in ≥ 4 (33%) of the NSCLC of patients with brain metastasis. This resulted in 108 variants in 20 genes (Figure 2 and Supplementary Table 1). The average number of unique variants per gene was 5.4, ranging from a low of 2 variants in SMAD4 to a maximum of 9 in BRCA2 and EP300. Among the 20 genes, 8 had variants in ≥ 50% of NSCLC with brain metastases. This included TP53 and CREBBP which had no unique variants in the controls. It should be noted that all specimens contained variants in TP53; however, these results indicate that 11 cases of NSCLC with brain metastasis had variants not found in any of the patients without brain metastasis.

IVA then identified the signaling pathways that were present in ≥ 10 cases of the patients with brain metastasis and ≤1 patients without brain metastasis. UVB-induced MAPK signaling, UVA-induced MAPK signaling, role of CHK proteins in cell cycle checkpoint control, JAK/STAT signaling and PI3K/AKT signaling were amongst the most prominent pathways altered in the BM patients.

### Development of a variant signature at the gene level to predict brain metastasis

**Figure 3:**
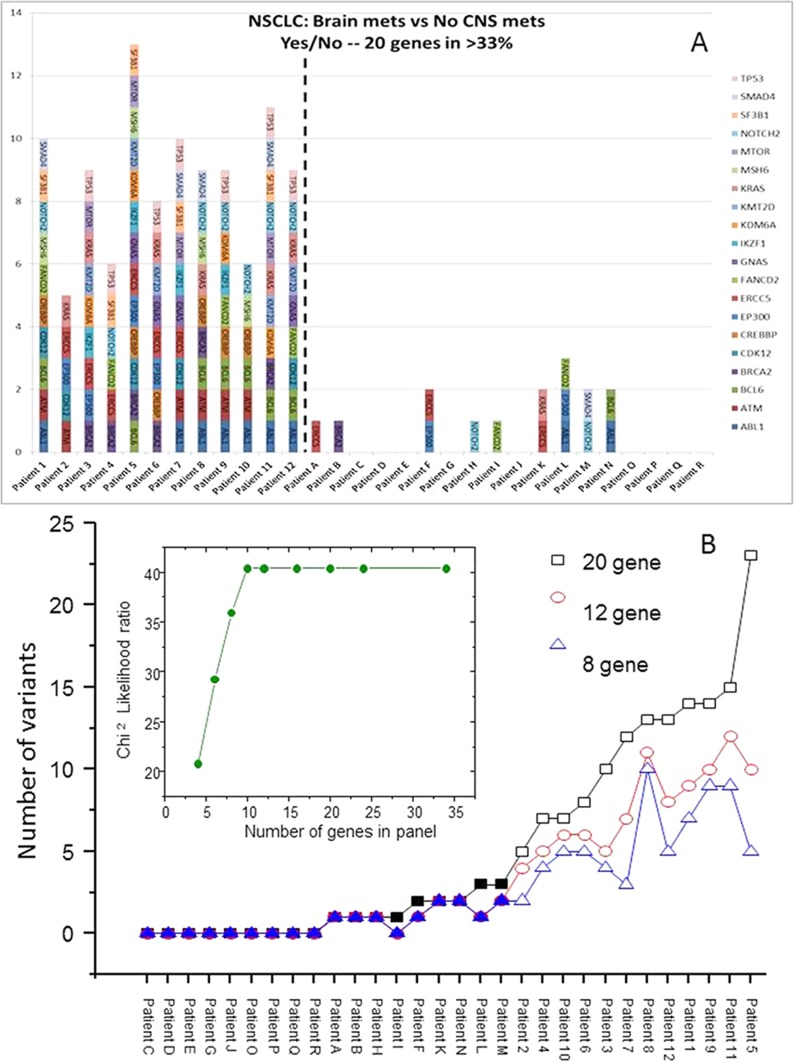
Variants present in individual tumors after filtering strategy In **(A)** the 20 most prevalent genes altered in the NSCLC with BMs are shown with the y-axis representing the number of variants in each gene. In **(B)** the patients have been ordered according to the number of variants in the 20-gene panel with additional data shown for 12 or 8 genes. The open symbols represent NSCLC with BMs. The inset shows the Chi^2^ Likelihood ratio for increasing number of genes.

The filtering strategy identified 167 variants in 34 genes in the NSCLC specimens from patients who developed a subsequent brain metastasis; data from 20 of the commonly altered genes are presented in Figure 2. To develop a predictive test, the data were analyzed at the patient level. Figure 3A shows the transform of Figure 2 where the number of variants in the 20 genes is presented for each individual patient. At the 20 gene level, there is a minimum of 5 variants in each of the NSCLC with BMs and a minimum of 5 genes with variants. The average number of genes with variants was 8.75, and the average number of variants was 10. Patient 5 exhibited the largest number of variants with 23 in 13 of the genes. In Figure 3B, we have investigated the minimum number of genes that may be required to develop a predictive test from this exploratory data. The genes were ordered in the prevalence of their mutation incidence amongst the NSCLC patients with BMs and analyzed for their significance to discriminate the two groups of patients using a log-rank test. Using the panel of the 8 most frequently altered genes (*TP53, BCL6, ABL1, NOTCH2, ATM, CREBBP, BRCA2* and *KRAS*) would result in three false positives (or negatives) as these were also the genes most commonly detected at the allowed filtering level in the non-BM NSCLCs. The inset shows the significance of the discrimination power plateaus at using the ten most commonly altered genes (which added *ERCC5* and *CDK12* to the panel); this panel of genes discriminates all patients with BMs from those without BMs.

### Mutation landscape of NSCLC brain metastases compared to primary NSCLC without brain metastasis

The focus then shifted to genomic variation in the actual brain metastases of NSCLC patients. The brain metastasis samples include those from NSCLC patients with synchronous brain metastasis (n=10) and those with interval brain metastasis (n=8). These were compared to the same cohort from the previous analysis of NSCLC patients without brain metastasis (n=18). Variants were filtered as described in Figure 1. Individual variant analysis identified only a single variant in >33% of the brain metastases. This variant, a C→T missense mutation in CDK12 (c.3811C>T/p.P1280S), occurs in 7 brain metastases but only 1 of the controls. While classified as a variant of unknown significance by ACMG guidelines, it is classified as “tolerated” using SIFT function predictions.

**Figure 4:**
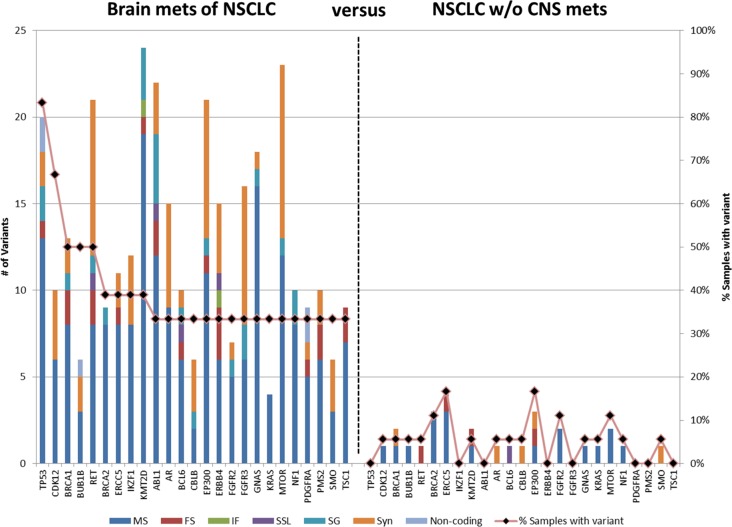
Genes containing variants in BMs of NSCLC patients Variants in > 6% NSCLC without CNS metastasis were filtered then genes were identified with variants in ≥ 33% of brain metastasis. MS: missense, FS, frameshift; IF, in-frame; SSL, splice site loss; SG, stop gain, Syn, synonymous. The bars represent number of variants; the line represents the percentage of specimens with variants in the gene.

Filters were then applied to determine genes that have variants in brain metastases but not in NSCLC patients without brain metastasis. Variants that occur in > 1 of the NSCLC samples were filtered; genes were then identified that that had variants in ≥ 6 (≥ 33%) of the brain metastases. This resulted in 327 variants in 25 genes (Figure 4 and Supplementary Table 2). The average number of unique variants per gene was 13.1, ranging from a low of 4 variants in KRAS to a maximum of 24 in KMT2D. Of note are two genes, TP53 and CDK12, which had unique variants in more than two-thirds of brain metastasis. TP53 had 20 variants spread across 15 cases of brain metastases (83%) whereas none of these 20 variants were present in a control. This includes 11 variants that are classified as pathogenic or likely pathogenic by ACMG guidelines. CDK12 demonstrated 10 unique variants in 12 cases of brain metastasis with only a single control containing one of these variants.

**Figure 5:**
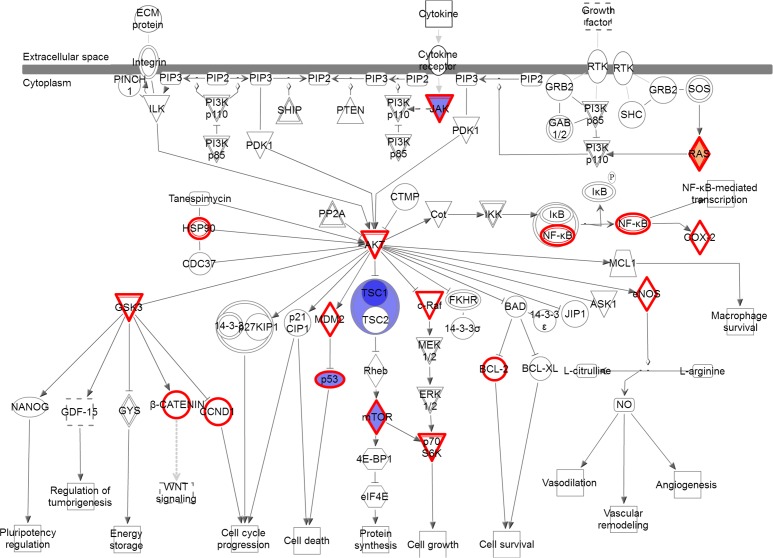
Pathways altered in brain metastases compared to primary NSCLC **(A)** the PI3K/AKT and JAK/STAT signaling pathways and **(B)** the DNA Damage Response pathway. Red = gain of function, Blue = loss of function; Gray = inferred normal. Entities outlined in red are potential drug targets.

No individual variant was present at ≥ 33% in both NSCLC primaries and brain metastases compared to NSCLC without brain metastasis. At the gene level, the number of genes and number of variants within those genes were higher in the brain metastases compared to the primary tumors of patients with brain metastasis (Figure 2 vs Figure 4). Additionally eleven genes were altered in both comparisons; however, only a single gene (TP53) was found to have variants in ≥ 50% of both the brain metastases and the NSCLC primary tumors with brain metastasis. Furthermore, certain signaling pathways were altered in a large majority of both the primaries and brain metastases. Pathways were identified that contained variants in > 90% of cases (either ≥ 11 of the 12 brain metastasis or ≥ 17 of 18 the primary NSCLC with brain metastasis) and ≤ 5% (≤ 1) of NSCLC without brain metastasis. These highly represented signaling pathways include “PI3K/AKT Signaling” and “JAK/STAT Signaling” (Figure 5A) which were represented by variants in ATM, JAK3, KRAS, and MTOR. All 11 of 12 cases contain at least 1 variant in 1 of those genes, while only 1 control has a variant (a single control with a variant in KRAS). Another key pathway was “Role of CHK Proteins in Cell Cycle Checkpoint Control” with variants involved in these pathways include ATM, BRCA1, KRAS, MTOR, and TP53 (Figure 5B).

## DISCUSSION

The genomic variants present in the tumors of patients with non-small cell lung cancer have been shown to have an effect on various aspects of tumor development and progression. Tumors with EGFR mutations are smaller and more aggressive than those without variants, and patients with variants more often develop distant metastases to lung and brain [30]. Some groups have found that, while EGFR status did correlate to overall survival, it did not increase the likelihood of metastasis to the liver [31]. Conversely, Doebele et al demonstrated that NSCLC with EGFR mutations as well as ALK gene rearrangements were more likely to develop liver metastasis [32]. However, this same group was unable to show a predisposition between a given mutation pattern and metastasis to adrenal gland, bone, or brain. These results demonstrate the necessity for further investigation into the role of genetic variation on the development and site of metastasis.

A study by Vignot et al compared genomic alterations in the archived primary tumors of NSCLC patients to alterations found in the patient-matched metastases [33]. The study included a variety of histologic types and metastatic sites. Their conclusions pose an interesting comparison to our current study which focused on a single histologic type (adenocarcinoma) and a single metastatic site (brain) even with the fact that the majority of our study consisted of samples that were not paired. Two KRAS variants (c.35G>A/p.G12D and 37G>T/p.G13C) identified in matched tumor-metastasis pairs were also found in both the primary tumors and brain metastasis of the current study. Additionally, Vignot et al found that 12 of the 15 (80%) matched pairs had TP53 variants with 10 patients containing more than one TP53 variant. We found more than 58% of the primaries and 83% of brain metastases contained unique TP53 variants. One variant (511G>T/p.E39^*^) was found in patients of both studies.

Furthermore, several other groups have identified TP53 as a driver of NSCLC. In a survey of the Catalogue of Somatic Mutations in Cancer (COSMIC) database, Zhang et al found gene mutations in nearly 40% of lung adenocarcinoma [34]. In a different study, TP53 demonstrated the most gene variations in lung adenocarcinomas including 30% of all non-synonymous base substitutions [35].

In addition to individual variants, we found that genes involved in PI3K/AKT signaling were highly altered in both the primary NSCLC and the brain metastasis with over 90% of patients containing a variant in this pathway. This correlates with findings from other researchers. In one study, alterations in the PI3K pathway were found in 33% of cases of NSCLC and included variants in AKT1, PIK3R2, and MTOR [35]. In a review of variants present in the COSMIC database, the MAPK and PI3K signaling pathways were identified as the main oncogenetic pathways involved in lung adenocarcinoma [34]. Li et al investigated brain metastasis and discovered single nucleotide polymorphisms in PI3K-AKT-mTOR signaling that were associated with brain metastasis in NSCLC patients [36]. Specifically they found that certain germline polymorphisms in AKT1 and PIK2CA were associated with higher risk of developing brain metastasis. The importance of PI3K signaling variants may prove to be significant as they are an important downstream signaling pathway of EGFR, and these variants may be an important mechanism to the acquired resistance often seen following treatment with EGFR-targeted therapies [37].

As the therapeutic benefit of current treatment modalities for BMs is limited, identification of potential new avenues for treatment of these lesions is of urgent importance. In particular discovering druggable targets that cannot be identified in primary tumors may result in precision medicine based on the information from metastases from individual patients. Our study has uncovered several potential avenues to explore new treatments as well as confirm some current opportunities. EGFR therapy is an ongoing area of research in BMs from NSCLC [18, 38] with novel approaches such as “pulsative” treatment being studied mainly based at known mutant gene mutations. Other strategies could include the use of agents such as dacomitinib which is a second generation, highly selective, small-molecule pan-HER inhibitor might be an alternative approach [2, 38, 39] to current small molecule inhibitors. Our studies suggested that PI3K/AKT, JAK/STAT and CHK1 signaling may offer alternative opportunities as therapeutic approaches. A phase II trial has studied the CHK1 inhibitor, LY2603618 in combination with pemetrexed in a single-arm, open-label non-randomized study of patients with advanced or metastatic non-squamous NSCLC without demonstrating extra clinical benefit [40]. However, it could be argued that this combination may be more effective in combination with radiation treatment (RT) to exploit the DNA damage response (DDR) in mutant P53 cells which are common in BMs [41]. JAK/STAT pathway inhibition has been studied in pre-clinical models of NSCLC and has been shown to overcome resistance to cisplatin [42] and to enhance the anti-tumor activity of cetuximab [43]. Alterations in the PI3K/AKT/MTOR pathway have been identified as driver mutations in primary NSCLC lung cancer but also to be known as the biomarkers of resistance to EGFR-tyrosine kinase inhibitors [44]. Although several Phase I/II studies have been carried out in trials including primary NSCLC none have addressed this pathway in metastatic disease and none have combined the agents with radiation which remains standard-of-care for BMs. In addition, no studies have combined the use of targeted agents and radiation in orthotopic models of NSCLC brain metastases.

One of the limitations of molecular targeted agents in the setting of CNS metastases is their ability to cross the blood-brain barrier (BBB) [45] which has been demonstrated in reduced levels of gefitinib and erlotinib in the cerebrospinal fluid (CSF) of patients with NSCLC [46]. Radiation treatment (RT) is considered as a feasible strategy to optimize drug delivery into brain by inducing BBB tight junction damage and lead to improved penetration [47, 48] although this has not been observed universally [49]. This may be dose-dependent and time-dependent as other researchers have demonstrated that the CSF-plasma ratio of gefitinib increased with increasing dose of whole brain radiation treatment (WBRT) peaking at 30 Gy [50] whilst Teng and colleagues [51] showed that blood-tumor barrier opening increased 2-4 weeks after RT. Whole brain radiation treatment has the potential to synergize with many different classes of molecular targeted agents [52] as well as to improve their uptake through the BBB and thus may offer hope for patients with CNS metastases.

Our study does have several limitations. The study was based upon retrospective archival formalin-fixed paraffin-embedded (FFPE) tissue samples. Formalin fixation is known to produce sequencing artifacts, particularly at low allele frequency [53]; however, the vast majority of tissue available for research studies as well as much of the current clinical testing is done on FFPE tissue so we believe the data presented here to be particularly relevant. Furthermore, normal DNA was unavailable for analysis thereby limiting the analysis of germline variation. Prospective studies will include collection of blood for germline analysis and cell-free DNA. Additionally, we were unable to get paired primary tumor/brain metastasis from all patients. Ideally this would have allowed us to make a more direct comparison of variants important for metastatic spread. However, Vignot et al found a high degree of concordance between the mutational spectra of tumor-metastasis samples from the same patient [33]. Regardless, matching samples would not eliminate variability caused by intratumor heterogeneity that has been found to impact the detection of driver mutations in NSCLC [54]. Also, our study was not able to detect ALK rearrangements. Another limitation of the study is the sample size. The sample size was restricted by the challenge to find paired samples of primary and brain metastases where often if synchronous brain metastases are present, the primary is often not resected. In addition, there is usually only biopsy material present (if at all) for subsequent brain metastases. More samples could have been analyzed in some of the groups but we wanted to keep the sample size even between the groups so as to not run into problems with unequal variance. These issues may have also introduced a selection bias as the samples obtained from brain metastases may include patients with aggressive cerebral metastatic disease and multiple metastases which were not candidates for surgical treatment. Future studies may need to compare resectable and unresectable brain metastases.

In conclusion, we have identified a panel of genes that begins to discriminate patients that will go on to develop brain metastasis. The strategy to develop the predictive test will be to analyze a blinded independent set of specimens using the same sequencing methods and filtering and assess the same variants and genes identified from our exploratory cohort of patients. The ultimate goal would be to employ the classifier to select prophylactic irradiation at presentation or consider using drugs targeting the altered pathways identified in our studies.

## MATERIALS AND METHODS

### Human research protection

The Beaumont Human Investigation Committee approved all work under an approved research protocol (IRB # 2014-177).

### Patient selection

Due to the requirement to have long-term follow-up to detect metastatic events, this study used retrospective material which meant that there were no normal tissue or blood samples available for analysis. The sample size was restricted by the challenge to find paired samples of primary and brain metastases. If synchronous brain metastases are present, the primary is often not resected. In addition, there is usually only biopsy material present (if at all) for subsequent brain metastases. Also, some of these patients may be treated for their brain metastases elsewhere. The sample size was dictated by being able to trace and find pathological material for the different groups which were studied. More samples could have been analyzed in some of the groups but we wanted to keep the sample size even between the groups so as to not run into problems with unequal variance. Archival formalin-fixed paraffin-embedded (FFPE) tumor samples with the diagnosis of non-small cell lung cancer were obtained from Beaumont Health Department of Pathology. Hematoxylin-eosin slides were reviewed by a single pathologist for identification of tumor location. Patients with NSCLC were selected using the following criteria: 1) no evidence of metastasis within 5 years of surgery (n=10); 2) metastatic disease but no CNS involvement (n=8); 3) brain metastasis subsequent to NSCLC diagnosis (n=12); and 4) simultaneous diagnosis of NSCLC and metastatic brain lesions (n=10). Patients with newly diagnosed non-small cell lung cancer included in this study had routine neuroimaging at the time of their initial diagnosis which was negative. Subsequent neuroimaging was ordered only if the patients developed symptoms concerning for brain metastases. We chose a 6-month minimum period between surgery and detection of brain metastases to minimize the possibility that the metastases were present but remained undetected at presentation. Table 1 describes the sample sites analyzed for each patient. All patients with synchronous brain metastases had only tissue from the brain lesion available for analysis. For the 12 patients with brain metastases subsequent to NSCLC diagnosis, 7 had both primary NSCLC and brain metastasis tissue available; 4, only the primary NSCLC; and 1, only the brain metastasis.

### DNA isolation

The tissue section used for isolation was a 1 mm core that was removed using a TMArrayer (Pathology Devices, Winchester, MD) from the previously marked tumor area. DNA was isolated using QIAmp DNA Micro Kit (Qiagen, Valencia, CA). Manufacturer's protocol was followed with a modified condition that included an RNase A treatment immediately following overnight incubation. Quality and amplifiable DNA material was assessed with the GeneRead DNA QuantiMIZE Kit (Qiagen, Valencia, CA).

### Library preparation

Targeted enrichment multiplex PCR of 160 genes was done using GeneRead DNAseq Comprehensive Cancer Panel V2 (Supplementary Table 3) in combination with GeneRead DNAseq Panel PCR Kit V2 (Qiagen, Valencia, CA) following manufacturer's protocol. Starting amount of amplifiable DNA used and PCR cycles for initial library amplification were based on calculation results determined by GeneRead DNA QuantiMIZE kit. Sample pooling and purification were subsequently done using Agencourt AMPure XP Beads (Beckman Coulter, Brea, CA). Library construction was done using GeneRead Illumina based DNA Library Prep Kits with sample barcode multiplexing done using GeneRead Adapter I Set 12-plex (Qiagen, Valencia, CA). The resulting barcoded constructed libraries were then quantified using GeneRead DNAseq Quantification Kit. Samples were pooled and sequenced using the NextSeq 500 (Illumina, San Diego, CA) as paired-end 150 base reads (2×150).

### Alignment

Results of sequencing runs were de-mutliplexed and FASTQ files generated in Illumina's BaseSpace. The FASTQ files were converted to FASTA format for analysis in NextGENe (SoftGenetics, State College, PA). In this conversion, reads were filtered to retain reads with median score threshold ≥ 10, maximum number of uncalled bases ≤ 3, and total bases called in a read ≥ 20, as well as reject reads when ≥ 3 bases with score ≤ 10. Alignment to v37 of the whole human genome was carried out in NextGENe using a Preloaded Index Alignment algorithm. This algorithm employs a suffix array that is represented by the Burrows-Wheeler Transform (BWT).

### Mutation analysis

Mutations were called by NextGENe based upon mutation percentage ≥ 5% and total coverage ≥ 250. The coverage requirement was ignored for homozygous mutations. For mutations occurring at a frequency ≤ 80%, the balance ratio of forward and reverse reads was required to be > 0.1. Further biological interpretation was done in Ingenuity Variant Analysis (IVA; “2016 Winter Release”; Qiagen, Redwood City, CA) software through a series of filtering steps (Figure 1). “Common Variants” were filtered by excluding variants with an allele frequency ≥ 1.0% in the 1000 Genomes Project, the NHLBI Grand Opportunity Exome Sequencing Project (GO ESP), the Allele Frequency Community, and the Exome Aggregation Consortium (ExAC). Variants were then filtered to include those predicted to be deleterious by including only those experimentally observed to be: Pathogenic, Possibly Pathogenic, associated with gain of function (as established in the literature or gene fusion), or associated with loss of function of a gene (via frameshift, in-frame indel, or stop codon change, or a missense mutation). For comparisons across groups, variants were retained that occur in ≥ 33% of the case samples and > 1 control samples. Controls were defined as patients that did not develop brain metastases; this included both patients without metastases and patients who developed non-CNS metastases. Variants were required to be associated with gain of function or heterozygous, haploinsufficient, heterozygous ambiguous, hemizygous, compound heterozygous, or homozygous. For gene level analysis, the filtering is the same as at the variant level; however, variants were first filtered if they occur in >1 control then a gene was required to have any variant in 33% of cases.

## SUPPLEMENTARY MATERIALS TABLES







## Abbreviations

NSCLC: non-small cell lung cancer
CNS: central nervous system
BM: brain metastasis
PCI: prophylactic cranial irradiation
SCLC: small cell lung cancer
EGFR: epidermal growth factor receptor
EML$: echinoderm microtubule-associated protein like 4
ALK: anaplastic lymphoma kinase
TCGA: the Cancer Genome Atlas
ACMG: American College of Molecular Genetics and Genomics
SIFT: Sorting Intolerant from Tolerant
COSMIC: Catalogue of Somatic Mutations in Cancer
DDR: DNA damage response
BBB: blood-brain barrier
CSF: cerebrospinal fluid
RT: radiation treatment
WBRT: whole brain radiation treatment
FFPE: formalin-fixed paraffin-embedded.
