# Supplementary material for: Targeted DNA sequencing of non-small cell lung cancer identifies mutations associated with brain metastases
Source: Oncotarget. 2018 May 25;9(40):25957–70. doi: 10.18632/oncotarget.25409 (PMC5995256; doi:10.18632/oncotarget.25409)
Supplement: Supplementary file 1 [file oncotarget-09-25957-s001.pdf]

Targeted DNA sequencing of non-small cell lung cancer identifies mutations associated with brain metastases

SUPPLEMENTARY MATERIALS

Supplementary Table 1: Variants that occurred in ≥ 4 of NSCLC of patients with brain metastases but ≤ 1 of the NSCLC samples without brain metastases

See Supplementary File 1

Supplementary Table 2: Variants that occurred in ≥ 6 of the brain metastases but ≤ 1 of the NSCLC samples without brain metastases

See Supplementary File 2

Supplementary Table 3: Genes included in the comprehensive cancer panel

ABL1	BUB1B	DDR2	FGFR2	IDH2	MEN1	PDGFRA	SMARCA4
AKT1	CARD11	DICER1	FGFR3	IKZF1	MET	PHF6	SMARCB1
AKT2	CBL	DNMT3A	FH	IL6ST	MLH1	PIK3CA	SMO
ALK	CBLB	ECT2L	FLCN	IL7R	MSH2	PIK3R1	SPOP
AMER1	CD79A	EGFR	FLT3	JAK1	MSH6	PMS2	SRC
APC	CD79B	EP300	FUBP1	JAK2	MTOR	PPP2R1A	STK11
AR	CDC73	EPCAM	GATA1	JAK3	MUTYH	PRDM1	SUFU
ARID1A	CDH1	ERBB2	GATA2	KDM6A	MYC	PRKAR1A	TERT
ARID2	CDK12	ERBB3	GATA3	KDR	MYD88	PTCH1	TNFAIP3
ASXL1	CDK4	ERBB4	GNA11	KIT	NF1	PTEN	TNFRSF14
ATM	CDKN2A	ERCC5	GNAQ	KLF6	NF2	PTPN11	TP53
ATRX	CHEK2	ESR1	GNAS	KMT2D	NFE2L2	RAC1	TSC1
BAP1	CIC	EZH2	GPC3	KRAS	NFKBIA	RB1	TSC2
BCL6	CREBBP	FAM46C	GRIN2A	MAP2K1	NOTCH1	RET	TSHR
BCOR	CRLF2	FANCA	H3F3A	MAP2K2	NOTCH2	ROS1	U2AF1
BRAF	CSF1R	FANCD2	HIST1H3B	MAP2K4	NPM1	SDHB	VHL
BRCA1	CTNNB1	FANCE	HNF1A	MAP3K1	NRAS	SETD2	WT1
BRCA2	CYLD	FAS	HRAS	MAP4K3	PALB2	SF3B1	XPC
BRIP1	DAXX	FBXO11	HSPH1	MDM2	PAX5	SLC7A8	ZNF2
BTK	DDB2	FBXW7	IDH1	MED12	PBRM1	SMAD4	ZRSR2
